# Effect of Ta Interlayers on Texture and Magnetic Properties of FeSi Films with Micrometer Thickness

**DOI:** 10.3390/ma15196789

**Published:** 2022-09-30

**Authors:** Jialian He, Zhong Zhang, Zhihao Bao, Guangai Sun, Xinxi Li, Xuepeng Qiu, Shiqiang Wang, Zhanshan Wang, Qiushi Huang, Shengzhen Yi

**Affiliations:** 1 MOE Key Laboratory of Advanced Micro-Structured Materials, Institute of Precision Optical Engineering (IPOE), School of Physics Science and Engineering, Tongji University, Shanghai 200092, China; 2 Shanghai Key Laboratory of Special Artificial Microstructure, Pohl Institute of Solid State Physics and School of Physics Science and Engineering, Tongji University, Shanghai 200092, China; 3 Key Laboratory of Neutron Physics, Institute of Nuclear Physics and Chemistry (INPC), China Academy of Engineering Physics (CAEP), Mianyang 621999, China

**Keywords:** FeSi film, Ta interlayer, saturation magnetization, coercivity, texture

## Abstract

Magnetized soft ferromagnetic films with micrometer thickness were studied. A FeSi film, with a total thickness of 2000 nm, separated by 10 nm-thick Ta interlayers, was fabricated using the direct-current magnetron sputtering technique. The thickness of each FeSi layer between adjacent Ta layers was 100 nm. Hysteresis loop measurement was used to characterize the magnetic properties of the layer. X-ray diffraction patterns and high-resolution transmission electron microscopy were used to characterize its texture. The experimental results showed that the FeSi film separated by Ta interlayers exhibited a lower saturation magnetization and a higher coercivity than those of the 1140 nm-thick FeSi film. The insertion of Ta interlayers resulted in the disappearance of the crystal plane of FeSi (221), and better texture of the crystal plane of FeSi (210). The FeSi film exhibited a crystal plane of FeSi (210) with a bcc crystalline structure. The Ta interlayers were partially amorphous, exhibiting crystal plane of Ta (002) and TaSi_2_ (310). The matching of magnetic properties between interlayers and soft magnetic layers played an important role in maintaining its soft magnetic properties.

## 1. Introduction

Soft magnetic materials can rapidly switch their magnetic polarization under a small, applied field, which have been widely used in inductors, power electronics, sensors, transformers, and electrical machines [1,2,3]. They are usually characterized by an intrinsic coercivity of less than 1000 A/m. Various effects on soft magnetic properties, such as the annealing temperature [4], seed layer [5,6], buffer layer [7], substrate material [8], and element additions [9], have been researched to improve the soft magnetic properties of soft magnetic thin films. Fe-based soft magnetic alloys have attracted interest in different research circles because of the comparable comprehensive source, high saturation induction, and notable direct current bias [10,11,12,13].

Xi et al. [14] reported that the degradation of the soft magnetic properties of FeNiSm films could be avoided by the insertion of a Ta interlayer, which may be used in other magnetic thin films when the degradation of the soft magnetic properties results from the growth of columnar grains. The in-plane hysteresis loop of a 200 nm FeNiSm sample with one 6 nm Ta interlayer exhibited a secondary overturning phenomenon because of the slightly different magnetic properties of the two separated FeNiSm layers. This phenomenon was also found in the magnetic hysteresis loop of a 2000 nm FeSi film separated by one Cr layer, as reported previously. Svalov et al. [15] reported that the nonmagnetic and magnetic spacers had different effects on the magnetic softness of FeNi films. Additionally, spacers were used to maintain the individual FeNi layer thickness below the critical thickness corresponding to a transition into a “transcritical” state. The aforementioned study was based on a sandwich structure, and the total thickness of FeNi was only 340 nm. However, the choice of interlayer material and the effect of interlayers on the magnetic properties were not clearly reported. Therefore, it is important to study the matching between the interlayers and soft magnetic layers.

Due to their wide range of uses in electrical equipment including transformers, high-speed motors, and power generators, FeSi alloys have recently gained a great deal of interest as soft magnetic materials [16]. Compared with permalloy, FeSi alloys exhibited a higher saturation magnetization, strongly depending on the chemical composition [17]. The soft magnetic properties of FeSi alloys deteriorate with increasing thickness similar to permalloy [18].

In this study, Ta interlayers were inserted into an FeSi magnetic thin film with a 2000 nm total thickness to avoid the degradation of its soft magnetic properties. When the monolayer thickness of FeSi film reached 1000 nm, its magnetic properties could no longer meet the requirements of the π-flipper. Therefore, we introduced interlayers to avoid the degradation of the magnetic properties of the FeSi film.

## 2. Materials and Methods

The FeSi film was separated by the insertion of Ta interlayers. The multilayer was fabricated using a direct-current magnetron sputtering technique on Si (100) substrates, which were 140 mm × 80 mm in dimension. Consequently, an FeSi film with a total thickness of 2000 nm was separated by 10 nm-thick Ta interlayers. The thickness of each FeSi layer between adjacent Ta layers was 100 nm. The base pressure prior to the deposition was below 8.5 × 10^−5^ Pa, and the Ar gas pressure with a purity of 99.999% was maintained at 0.133 Pa during the whole sputtering process. The FeSi alloy and Ta sputtering targets had diameters of 101.6 mm. The sputtering target of Ta had a material purity of 99.999%. The FeSi target had a mass percentage of Si around 6.5 wt%. The sputtering distance of FeSi alloy and Ta targets were set at 8 and 6 cm, respectively. The substrate was moved over the spattering region of each target for layer coating during the deposition process. The magnetron cathodes were operated in a power constant mode while the powers were set at 40 W for FeSi alloy and 30 W for Ta targets. As the deposition rates were fixed for both targets, the varied thickness of each layer was achieved by varying the moving speed of the substrate across its sputtering region during the whole sputtering process. In this study, the moving speed of the substrate for FeSi layers was 0.35 mm/s, and 0.23 mm/s for Ta layers.

The magnetic properties of the FeSi layers were studied using a vibrating sample magnetometer (Quantum Design PPMS-9 T system). Their texture was characterized via X-ray diffraction (XRD) using a Bruker D8 diffractometer with a five-axis configuration and Cu Kα radiation (λ = 0.1542 nm). Transmission electron microscopy (TEM) was used to determine the multilayer thickness. The high-resolution transmission electron microscopy (HRTEM) analysis in this study was supported by Materials Analysis Technology Inc. (MA-tek). The HRTEM micro-analysis was conducted using the FEI G2 F20 Tecnai, which was operated at 200 keV.

## 3. Results and Discussion

### 3.1. Hysteresis Loop Measurement

**Figure 1:**
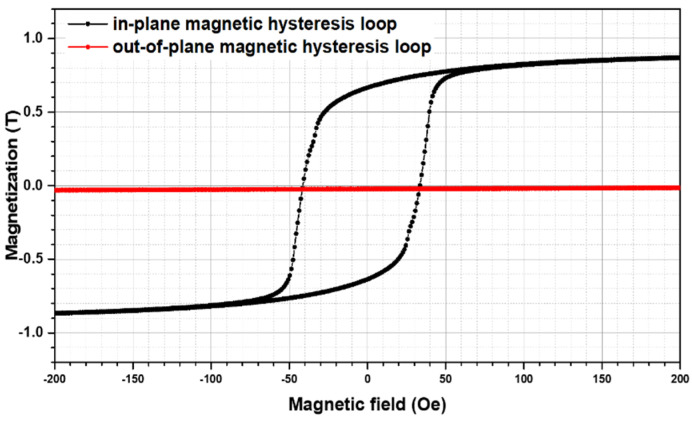
Magnetic hysteresis loop of the FeSi film separated by Ta interlayers in-plane (black) and out-of-plane (red).

The experimental magnetic hysteresis loop of the FeSi film separated by Ta interlayers is depicted in Figure 1. The thickness of the FeSi layer was characterized using TEM. 

For the application of FeSi films in π-flippers, films must be saturated over 1 T in a small external magnetic field to meet the requirements of the larger spin echo length. The FeSi film separated by the Ta interlayers exhibited a saturation magnetization (*M_s_*) of 0.8 T and coercivity (*H_c_*) of 37.5 Oe. The film had a lower *M_s_* and higher *H_c_* than the 1140 nm-thick FeSi monolayer, exhibiting a *M_s_* of 1 T and a *H_c_* of 10.3 Oe. Therefore, with the insertion of Ta interlayers, the soft magnetic properties of FeSi film were not improved.

### 3.2. X-ray Diffraction Measurement

**Figure 2:**
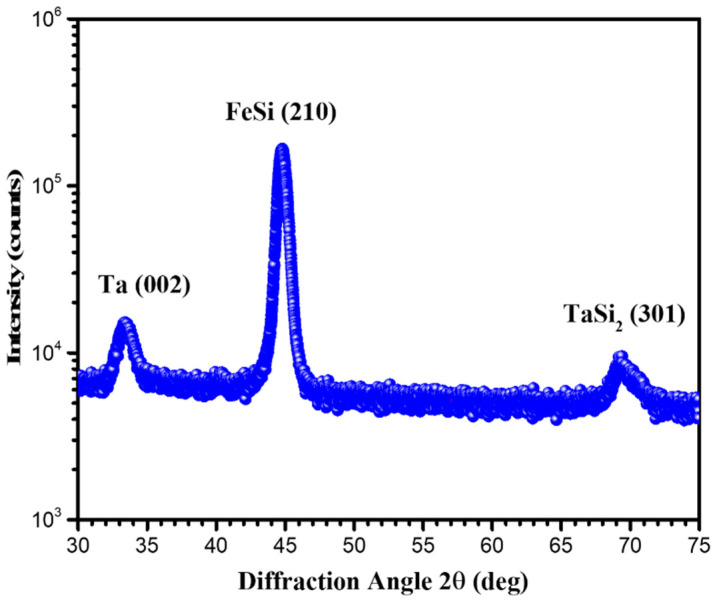
XRD profile of the FeSi film separated by Ta interlayers.

The XRD measurement shown in Figure 2 revealed that the Ta interlayers exhibited a crystal plane of Ta (002) at 33.692° and TaSi_2_ (301) at 69.512°. It can be seen that the peak that appeared around 45.124° could be identified as the (201) diffraction peak of the nano-crystalline FeSi grains.

The full-width at half-maximum (FWHM) of Ta (002) was 1.314° with a grain size of 63 Å, and the FWHM of FeSi (210) was 0.727° with a grain size of 119 Å. The FeSi film separated by Ta interlayers exhibited the only crystal plane of FeSi (210), which is different from the FeSi film separated by Cr interlayers exhibiting crystal plane of FeSi (210) and FeSi (221) [19]. Their soft magnetic properties were totally different, which could probably be explained by the different texture of the FeSi film.

### 3.3. High Resolution Transmission Electron Microscopy

**Figure 3:**
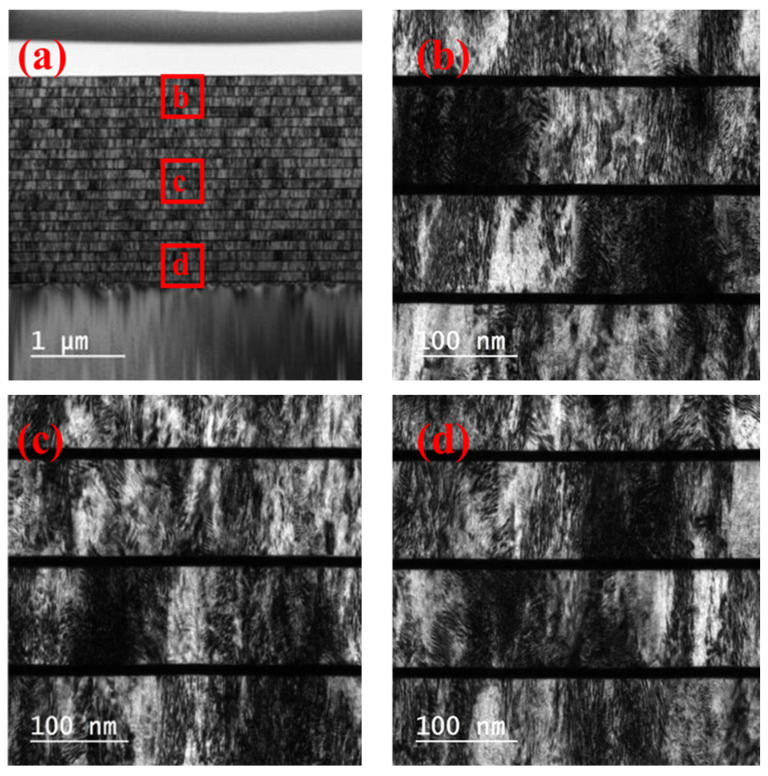
Transmission electron microscopy results for FeSi film separated by Ta interlayers. (**b**), (**c**,**d**) Representing higher resolution images of the top, medium, and bottom of the red areas in (**a**), the whole image, respectively.

The full view image of the sample, including the Si substrate and the passivation layer at the top, is shown in Figure 3a using the bright field mode in TEM. Figure 3a–d represents higher resolution images of the top, medium, and bottom of the red areas. The bright layers are FeSi, and the dark layers are Ta. The FeSi layers showed a columnar structure with large column grains perpendicular to the film plane. It was observed that the growth of the FeSi grains was discontinuous.

**Figure 4:**
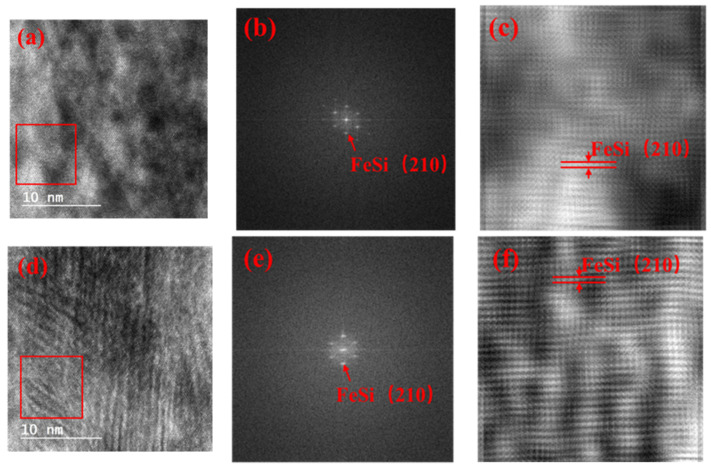
High-resolution transmission electron microscopy results of (**a**) the top and (**d**) bottom FeSi layers. (**b**,**c**) Representing Fast Fourier Transform (FFT) and inversed FFT images of the red area enclosed in the box in panel (**a**), respectively, and (**b**) showing various spots of FeSi. (**e**,**f**) Representing FFT and inversed FFT images of the red area enclosed in the box in panel (**d**), respectively, and (**e**) showing various spots of FeSi.

HRTEM images of the top and bottom FeSi layers are shown in Figure 4a,d, respectively. To further understand the HRTEM results, the Fast Fourier Transform (FFT) and inversed FFT images were extracted from the red area enclosed in the box in panel (a). Figure 4b,e shows that the FeSi layer exhibited a polycrystalline state. The crystal plane of FeSi (210) was found in the inversed FFT images, which was consistent with the result of XRD.

**Figure 5:**
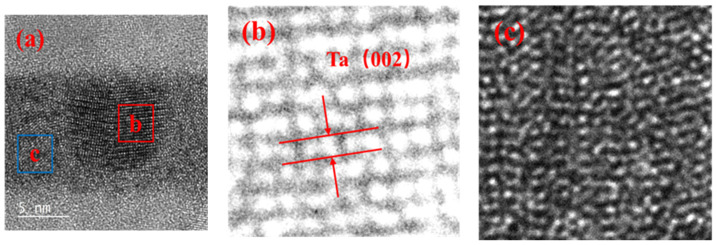
(**a**) High-resolution transmission electron microscopy results of the Ta interlayer (**b**) and (**c**) representing inversed FFT images of the red and blue areas in (**a**), respectively.

High-resolution transmission electron microscopy results of the Ta interlayer performed on cross-sections showed small grains embedded in an “amorphous like” layer, as shown in Figure 5. This suggested that the Ta interlayers were partially amorphous, and the crystallization plane of this interlayer was Ta (002) with a tetragonal crystal structure. The preferred orientation of TaSi_2_ was not found. It is possibly because the few TaSi_2_ mainly exist in the interfaces. The crystallization state of Ta interlayers was probably related to the melting point value 2996 °C of Ta. This should affect the atomic surface mobility during the growth and influence the microstructure. Lower atomic mobility was expected in the Ta interlayers, inhibiting the crystallization process. This probably explained the partially amorphous structure of the Ta interlayers.

## 4. Conclusions

The experimental magnetic hysteresis loops showed that the FeSi film separated by Ta interlayers exhibited a lower saturation magnetization and higher coercivity compared to the result of 1140 nm-thick FeSi monolayers. The XRD experimental results showed that the FeSi film separated by Ta interlayers exhibited obvious FeSi (210), while Ta (002) and TaSi_2_ (310) were found in the Ta interlayers. The TEM experimental results demonstrated that the growth of FeSi grains was discontinuous. The FFT and inversed FFT images of the HRTEM experimental results showed that the Ta interlayers were partially amorphous with nano-crystalline Ta (002) and TaSi_2_ (301) grains. The insertion of Ta interlayers of FeSi film exhibited the crystal plane of (210), while the (221) was restrained. Ta interlayers could divide the FeSi films into separate layers below the critical thickness, and block the columnar growth of FeSi film with micrometer thickness. Therefore, the formation of the “transcritical” state was avoided.

The matching of magnetic properties between interlayers and soft magnetic layers played an important role in maintaining its soft magnetic properties. In this way, the method of interlayers to maintain the magnetic properties of soft magnetic films with increasing thickness was developed, and may be used in other magnetic films. 

## Data Availability

The data presented in this study are available on request from the corresponding author.
